# Q-Switched 1064/532 nm Laser with Nanosecond Pulse in Tattoo Treatment: A Double-Center Retrospective Study

**DOI:** 10.3390/life11070699

**Published:** 2021-07-16

**Authors:** Giovanni Cannarozzo, Steven Paul Nisticò, Elena Zappia, Ester Del Duca, Eugenio Provenzano, Cataldo Patruno, Francesca Negosanti, Mario Sannino, Luigi Bennardo

**Affiliations:** 1 Unit of Dermatology, Tor Vergata University, 00133 Rome, Italy; drcannarozzo@gmail.com (G.C.); dr.mariosannino@gmail.com (M.S.); 2 Department of Health Sciences, Magna Graecia University, 88100 Catanzaro, Italy; elena.zappia@studenti.unicz.it (E.Z.); ester.delduca@studenti.unicz.it (E.D.D.); cataldo.patruno@unicz.it (C.P.); luigi.bennardo@studenti.unicz.it (L.B.); 3 Unit of Dermatology, Mariano Santo Hospital, 87100 Cosenza, Italy; eprovenzano0@gmail.com; 4 Dermatologic Center “Villa Bella—Antiaging Care Group”, 40126 Bologna, Italy; francesca.negosanti@gmail.com

**Keywords:** Q-Switched laser, tattoo removal, laser, skin

## Abstract

Tattoo removal is a well-established procedure in dermatology. Lasers represent the gold standard in the management of this condition nowadays. In this study, we report our experience on the use of a Q-switched nanosecond source. A total of 52 patients were consecutively enrolled in performing tattoo removal at Magna Graecia University of Catanzaro and Tor Vergata University of Rome. Black and blue tattoos were treated with a 1064 nm laser, with a pulse duration of 6 ns and a fluence up to 10 J/cm^2^, while colored tattoos were treated with sessions of 532 nm laser, with a pulse duration of 6 ns and a fluence up to 5 J/cm^2^. Up to nine treatments with a minimum interval of 8 weeks between each session were performed. A six-month follow-up visit assessed patient satisfaction (Visual Analogue Scale). Overall clinical result was assessed with a clinical evaluation by two blinded dermatologists using a 5-point scale, comparing pictures before treatment and at follow up. A total of 52 patients were included and analyzed: 30 females (57.7%) and 22 males (42.3%). Mean age was 43.7 ± 12.7 years. According to Fitzpatrick’s skin classification, 16 individuals (30.8%) were type II, 15 (28.8%) were type III, and 21 (40.4%) were type IV. Most of the treated tattoos were carried out by professionals. The mean number of sessions required to obtain a result was 4.6 ± 2.5, and the final tattoo removal rate was 60% or higher, with 51.9% of the patients reporting highest satisfaction scores Q-Switched 1064/532 nm laser may be considered today as the gold-standard treatment for tattoo removal. Our results confirm literature findings of the safety and effectiveness of these devices.

## 1. Introduction

Tattooing, which involves creating a permanent skin pigmentation, has become a common phenomenon and a sign of individualism [1]. Individuals with tattoos sometimes seek the removal of tattoos for various reasons, becoming a more and more common request of patients in dermatological studies [2,3]. Although various methods have been proposed, such as surgery or chemical destruction, laser treatments are considered the most effective solution. Various lasers have been proposed to manage this condition. Traditionally, ablative lasers, such as CO_2_ lasers, are used [4].

However, this kind of laser induces superficial tissue destruction, therefore risk of scarring and hypopigmentation in tattoo treatment is very high [5,6]. For this reason, lasers selectively acting on specific chromophores, such as melanin or hemoglobin, have been proposed. 

The medical literature indicates that Nd YAG lasers acting at a 1064/532 wavelength are very effective in managing this condition [7,8].

Furthermore, different studies show that Q-Switched lasers release high energies in a minimal amount of time (nano or picoseconds). They also act selectively on tattoo pigmentation, sparing surrounding tissues [9,10,11]. This study aims to evaluate the safety and effectiveness of tattoo removal by using a Q-Switched 1064/532 nm laser treatment using a nanosecond pulse range.

## 2. Materials and Methods

All patients presenting for tattoo removal from the 1 January 2018 to the 30 December 2019 at the dermatological clinics of Magna Graecia University (Catanzaro, Italy) and University of Tor Vergata (Rome, Italy) were retrospectively enrolled in this study.

Exclusion criteria were the following: hypersensitivity to light (visible and near-infrared); medication known to increase sensitivity to light; therapies with anticoagulants and/or immunosuppressants; pregnancy or nursing; personal or family history of skin cancer; sun exposure in the three weeks before treatment (for any skin type); previous tattoo removal treatment; gold-containing medication; and recent exfoliation treatments, surgical treatments, and past skin disorders (including keloids). All patients signed informed consent on the risk of the procedure.

Patients included in the study were treated with a Q-Switched 1064/532 nm laser system (SmartPico^©^, Deka M.E.L.A., Calenzano, Italy), which provides ultrashort pulses to achieve selective photothermolysis of the target (tattoo pigments) with minimum thermal damage to surrounding biological structures. The physician chose treatment parameters according to skin phototype, tattoo class, and location. According to their characteristics (e.g., pigments, depths, etc.) treated tattoos were stratified according to the classes listed in Table 1 and their anatomical location, as per the Kirby–Desai scale [12].

Black and blue tattoos were treated with a 1064 nm laser, with a pulse duration of 6 ns and a fluence up to 10 J/cm^2^, while colored tattoos were treated with sessions of 532 nm laser, with a pulse duration of 6 ns and a fluence up to 5 J/cm^2^. Tattoos displaying black and another color simultaneously were treated with both wavelengths, according to the areas of interest. Up to nine sessions were performed in order to treat patients. The minimum interval between laser treatments was eight weeks, and it was increased to 12 weeks after the fourth session; appropriate scheduling is needed to achieve the physiological healing process, such as pigment removal and skin recovery, and also in order to lower the risk of side effects, such as “ghosting” of the removed tattoo. Patients’ satisfaction was considered the endpoint of treatments by physicians. Follow-up visits took place six months after the last laser session.

Before the first session, clinical photographic documentation was carried out and repeated six months after the last session. Pictures were taken using the same camera (Nikon 5600d, Nikon Corporation, Minato City, Tokyo, Japan) and parameters, the same shooting settings, a twin flash, and the same ambient light.

Two independent dermatologists evaluated the pictures, providing the final result in tattoo removal with a score in a 5-point scale (0–20% removal = 0; 20–40% removal = 1; 40–60% removal = 2; 60–80% removal = 3; 80–100% removal = 4).

**Figure 1:**
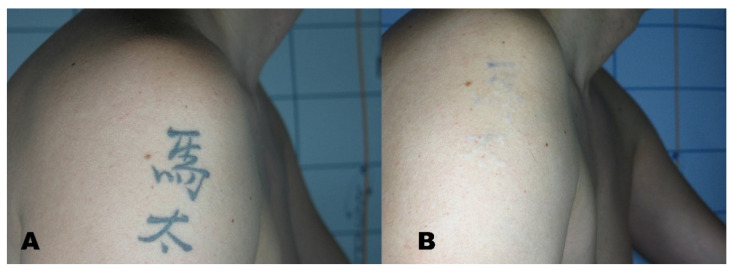
(**A**) Tattoo before treatment and (**B**) region after tattoo removal.

**Figure 2:**
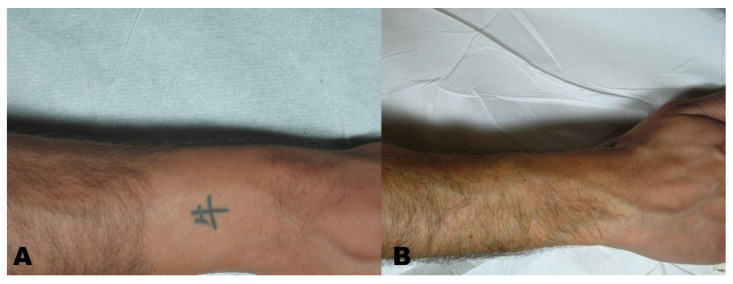
(**A**) Tattoo before treatment and (**B**) region after tattoo removal.

**Figure 3:**
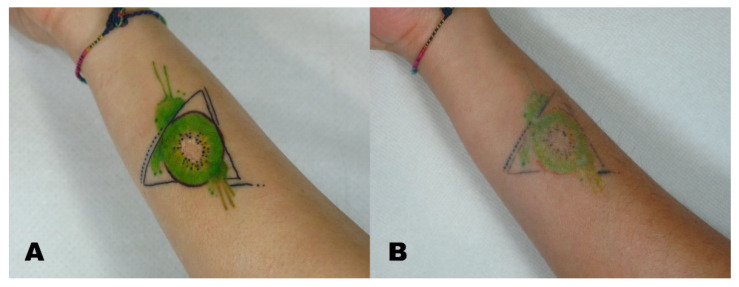
(**A**) Tattoo before treatment and (**B**) region after tattoo removal.

**Figure 4:**
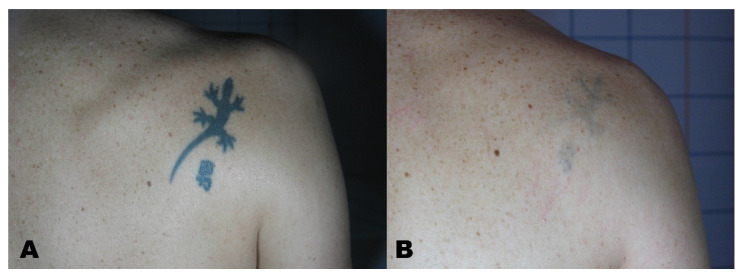
(**A**) Tattoo before treatment and (**B**) region after tattoo removal.

A Visual Analogue Scale (VAS) from 1 to 10 was administered to the patients at the six-month follow-up to measure patient satisfaction (Figure 1, Figure 2, Figure 3 and Figure 4).

Data analysis (mean, standard deviations, and rate calculations) was performed using Statistica 14.0 (TIBCO Software, Palo Alto, California).

## 3. Results

A total of 52 patients were included in this study and analyzed: 30 females (57.7%) and 22 males (42.3%). Mean age was 43.7 ± 12.7 years. According to Fitzpatrick skin classification, 16 individuals (30.8%) were type II; 15 (28.8%) were type III; and 21 (40.4%) were type IV. Most tattoos had been performed by professionals (*n* = 25–48.1%) and were characterized by intense pigments in dark, red, or mixed colors; other represented classes were amateur tattoos (*n* = 9–17.3%), followed by medical, traumatic (*n* = 7–13.5% each), and cosmetic ones (*n* = 4–7.7%). Tattoos were mainly placed on upper trunk/shoulder (*n* = 18–34.6%), lower arm/leg (*n* = 12–23.1%), and face/head (*n* = 11–21.2%), with fewer cases on wrist/hand/ankle/foot (*n* = 8–15.4%) or lower trunk/upper leg (*n* = 3–5.8%). Patients characteristics are reported in Table 2.

The number of laser sessions ranged from 2 to 9, with a mean number of 4.6 ± 2.5 (Table 3). The mean number of sessions was similar, considering different skin phototypes, while some qualitative trends could be observed in subgroups by tattoo type or location (Table 4); professional tattoos required more treatments, especially the ones with mixed colors, and the mean number of sessions increased moving from the head/face towards extremities. In all included patients, final tattoo removal rate evaluated by the two independent dermatologists was 60% or higher, i.e., score >3 (Table 3); more than half of cases (51.9%) were assessed with a top score (80–100% removal rate).

Considering the different subgroups, the best results were observed in phototype II and IV patients (score = 4 in 62.5% and 57.1% of cases, respectively), while in phototype III only one-third of cases (33.3%) reached 80–100% tattoo removal (Table 4). All cosmetic tattoos and 50.0–55.6% of professional tattoos were removed (an 80/100% clearance), while only 28.6% of medical ones received such a score. No explicit differentiation of removal results could be associated with different tattoo locations. All the patients reported high satisfaction after laser treatment; VAS score, 7.63 ± 1.53.

No serious adverse event occurred, and treatment was well tolerated by all patients. In two cases, a final “ghost effect” was observed; seven patients developed petechiae after session treatments and were medicated with an occlusive dressing, antibiotics, and hydrolytic enzymes, resolving the condition in 10–15 days.

## 4. Discussion

Laser interacts with tissues mainly thanks to three effects: the photothermal effect, characterized by conversion of the energy conveyed by a laser in heat, the photochemical effect, which breaks chemical bonds inside the pigment, and the photoacoustic effect, characterized by the mechanical destruction of the pigment. If wavelength, soot size, and fluence are correct, this will result in ink fragmentation and sparing of surrounding tissue. Whitening of the tissue right after laser treatment represents dermal and epidermal vacuolization due to localized steam formation, with signs of epidermal damage. [13,14]

This interaction creates newly generated unknown chemical compounds that may be disposed from the skin via the lymphatic system and generate a reaction from the immune system. Molecules remaining in the dermis may have different physical and biological characteristics. Metal-containing pigment could theoretically transform into toxic chemicals after light exposure, and cleavage of azo dyes may result in carcinogenic amines [13,15].

Colorants are the most important part of tattoo inks, consisting of almost insoluble pigments dispersed in water plus additives. A significant problem for the toxicological assessment of tattoo inks is the absence of appropriate data for ink composition and toxicology. Most tattoos are black, made by soot-related compounds (e.g., carbon black) with shading additives such as titanium dioxide or iron oxides. Potentially genotoxic polycyclic aromatic hydrocarbons can be found in skin specimens and regional lymph nodes even years after tattooing [14]. The same findings should apply to azo or polycyclic compounds in colored tattoos, as the pigments were designed for non-human use. Tattoo chromophores are mainly metal related. Aluminum, barium, copper, iron, strontium, and other metals with a lower safety profile, such as manganese, lead, and vanadium, have also been reported. Furthermore, many colorants may contain problematic substances such as primary aromatic amines, nitrosamines, metal pigments, phenols, formaldehyde, or phthalates. Some inks, such as red biolip, have been proved to have solid cytotoxic potential [13,14,15].

The QS Nd: YAG laser is to date considered a valuable tool for tattoo removal because its technology enables the obtainment of good results with minimal damage to the epidermis, dermis, and skin appendages, with a low risk of scarring and hypopigmentation [16,17,18]. An interval of at least two months between each session to allow complete pigment removal by macrophages and skin healing is recommended. Preoperative assessment must include evaluating the presence of scars under the tattoo, as it is mandatory to let the patient know that removing the tattoo would expose the underlying scar [19,20,21]. Despite the new advancement in tattoo removal, Q-Switched lasers may not be able to remove professional tattoos altogether; our study reported a complete clearance ratio in only 50% of patients, confirming what was already reported in the medical literature. Patients, especially when dealing with professional tattoos, should be informed of the risk of residual pigmentations [22,23]. Other side effects the patients should be aware of include hyperpigmentation, hypopigmentation, and skin darkening. The medical literature suggests that colored tattoos, especially green and yellow ones, respond poorly to laser removal, as do greater pigment density, larger tattoos (>30 cm), tattoos on the lower extremities, and those older than three years [18,24].

## 5. Conclusions

Our study confirms the results previously obtained in the medical literature, confirming Q-Switched laser treatments as the gold-standard treatment for tattoo removal. In our cohort of patients, we found that professional tattoos with mixed colors and tattoos on the lower limbs were more challenging to treat, while amateur tattoos and tattoos on the upper part of the body were more easily removed.

Of course, a prospective study with a more significant number of patients would be helpful to better analyze all the possible side effects and confirm our paper’s results.

## Figures and Tables

**Table 1:** Tattoo classes identified and included in the study.

Class	Specifications
Dark group (professional)	Performed by qualified personnel using mostly black-colored ink compositions
Red group (professional)	Performed by qualified personnel using ink compositions with predominantly red-colored pigments
Mixed color group (professional)	Performed by qualified personnel using multiple color and ink compositions
Amateur	Performed by unqualified personnel and usually lighter than professional tattoos
Medical	In cases of radiotherapy, usually dark blue in color, relatively superficial
Traumatic (natural)	Caused by various agents, with an unknown composition
Cosmetic (permanent makeup)	Performed by expert personnel using a mixture of components that often contain ferric oxide

**Table 2:** Demographic data of included patients.

Patient No.	52
Female (%)	30 (57.7%)
Male (%)	22 (42.3%)
Mean age ± SD (years)	43.7 ± 12.7
Age range (years)	23–62
*Fitzpatrick phototype:*	
II (%)	16 (30.8%)
III (%)	15 (28.8%)
IV (%)	21 (40.4%)
*Tattoo type:*	
Professional (%) out of which:	25 (48.1%)
Dark group (%)	9 (17.3%)
Red group (%)	4 (7.7%)
Mixed color group (%)	12 (23.1%)
Amateur (%)	9 (17.3%)
Medical (%)	7 (13.5%)
Traumatic (%)	7 (13.5%)
Cosmetic (%)	4 (7.7%)
*Tattoo location:*	
Face/head (%)	11 (21.2%)
Upper trunk/shoulder (%)	18 (34.6%)
Lower trunk/upper leg (%)	3 (5.8%)
Lower arm/leg (%)	12 (23.1%)
Wrist/hand/ankle/foot (%)	8 (15.4%)

See Table 1 for class specifications.

**Table 3:** Removal rate evaluation.

	No. Patients (%)	Mean No. Sessions ± SD	No. Sessions Range
Total	52 (100%)	4.6 ± 2.5	2–9
*Tattoo removal score*			
3 (60–80%)	25 (48.1%)	4.7 ± 2.6	2–9
4 (80–100%)	27 (51.9%)	4.6 ± 2.5	2–9

**Table 4:** Clinical parameters and removal scores in different subgroups.

	Mean No. Sessions ± SD	No. Sessions Range	Tattoo Removal Score 3 (60–80%) (*n*; %)	Tattoo Removal Score 4 (80–100%) (*n*; %)
*Fitzpatrick phototype:*				
II	4.6 ± 2.9	2–9	6 (37.5%)	10 (62.5%)
III	4.3 ± 2.5	2–8	10 (66.7%)	5 (33.3%)
IV	4.9 ± 2.3	2–9	9 (42.9%)	12 (57.1%)
*Tattoo Type:*				
Professional:				
Dark group	6.3 ± 0.9	5–8	4 (44.4%)	5 (55.6%)
Red group	6.5 ± 0.6	6–7	2 (50.0%)	2 (50.0%)
Mixed color group	7.8 ± 1.1	6–9	6 (50.0%)	6 (50.0%)
Amateur	2.3 ± 0.5	2–3	4 (44.4%)	5 (55.6%)
Medical	2.3 ± 0.5	2–3	5 (71.4%)	2 (28.6%)
Traumatic	2.6 ± 0.5	2–3	4 (57.1%)	3 (42.9%)
Cosmetic	2.3 ± 0.5	2–3	0 (0.0%)	4 (100.0%)
*Tattoo location:*				
Face/head	2.5 ± 0.5	2–3	4 (36.4%)	7 (63.6%)
Upper trunk/shoulder	4.2 ± 2.0	2–7	10 (55.6%)	8 (44.4%)
Lower trunk/upper leg	5.7 ±2.3	3–7	2(66.7%)	1 (33.3%)
Lower arm/leg	5.3 ± 2.5	2–8	6 (50.0%)	6 (50.0%)
Wrist/hand/ankle/foot	7.0 ± 2.9	2–9	3 (37.5%)	5 (62.5%)

See Table 1 for class specifications.

## Data Availability

Data are available from the corresponding author upon reasonable request.
